# Blue-Light-Driven Aerobic Oxidation via ROS-Generating Binuclear Cobalt(II) Complex Photocatalyst

**DOI:** 10.3390/nano16130835

**Published:** 2026-07-07

**Authors:** Yuhao Mu, Zhuang Miao, Rong Zhang, Xiong-Feng Ma, Zhipeng Xie

**Affiliations:** 1 College of Engineering, Xi’an International University, Xi’an 710077, China; xaiu24313@xaiu.edu.cn (Y.M.); miaozhuang@xaiu.edu.cn (Z.M.); xaiu24203@xaiu.edu.cn (X.-F.M.); 2 Department of Chemistry, The Chinese University of Hong Kong, New Territories, Hong Kong 999077, China

**Keywords:** binuclear complex, superoxide radical, blue light, aerobic oxidation, photocatalysis

## Abstract

Developing earth-abundant photocatalysts that operate efficiently under visible light remains a central challenge in sustainable aerobic oxidation chemistry. We synthesized a binuclear cobalt(II) structure (**Co_2_**) in which two redox-active metal centers are bridged by a polypyridine scaffold to integrate light-harvesting and catalytic functions within a single low-nuclearity unit. The complex exhibits a strong absorption band below 450 nm, undergoes facile charge separation upon photoexcitation, and channels molecular oxygen (O_2_) toward superoxide radical anion (O_2_^•–^) under blue-light irradiation. Spectroscopic and mechanistic studies indicate that the polypyridine framework governs photon capture and excited-state delocalization, whereas the proximal Co(II) sites mediate the subsequent single-electron transfer to O_2_. Driven by this dual-site synergy, **Co_2_** selectively oxidizes a broad scope of thioethers to the corresponding sulfoxides in yields exceeding 95%, with no over-oxidation to sulfones detected. The catalyst retains its structural integrity over five successive runs without measurable activity loss. By confining complementary photophysical and redox functions within a discrete bimetallic unit, this work establishes a design strategy for noble-metal-free, visible-light-driven organic transformations.

## 1. Introduction

Although solar energy holds significant potential as a sustainable energy source, the cost of photoabsorbing materials remains a major obstacle to its widespread application [1,2,3,4,5]. Anchoring light-absorbing molecules to the surface of semiconductors to enhance their light absorption capabilities offers an attractive advantage in constructing low-cost, practical devices from inexpensive and abundant semiconductor materials [6,7,8,9,10,11]. This research approach is also applicable to the design and synthesis of catalytic and photochemical materials for efficient solar energy utilization, enhancing their sustainability [12,13,14,15,16]. Therefore, an ideal photosensitizer complex needs to have a broad range of light absorption, which is crucial for achieving effective photoelectric conversion and injecting energy into catalytic reactions, or generating high concentrations of reactive excited states in catalytic reactions.

Photosensitizers capable of harvesting visible light and generating long-lived excited states are central to photochemical and photovoltaic applications. Ruthenium (Ru) and iridium (Ir) polypyridyl complexes have long served as benchmarks in this regard [17,18], with well-established MLCT excited states underpinning their use across photoredox catalysis, light-emitting devices, and solar energy conversion. Their scarcity and high cost, however, impose practical constraints on large-scale deployment, driving sustained interest in earth-abundant alternatives. Complexes based on copper (Cu), iron (Fe), cobalt (Co), chromium (Cr), and osmium (Os) have demonstrated competitive photosensitizing properties through targeted ligand design [19,20,21], and among first-row transition metals, Mn, Fe, Co, nickel (Ni), Cu, and zinc (Zn) have consequently emerged as priority targets for sustainable photosensitizer development [22,23]. Co(II) occupies a particularly attractive position within this landscape. When coordinated to π-conjugated polypyridine ligands, Co(II) can access MLCT and LLCT manifolds comparable to those of noble-metal benchmarks at substantially lower cost [24,25,26,27]. Beyond economics, Co(II) centers possess redox potentials and spin-state flexibility well matched to single-electron O_2_ activation (O_2_ → O_2_^•–^), a key step that remains less accessible for several other first-row metals under mild visible-light conditions. Peripheral ligand design plays a decisive role here: extended conjugated frameworks broaden light absorption and enhance LMCT/MLCT coupling, improving overall photoelectric conversion efficiency [27,28,29,30]. Compared to heterogeneous semiconductor photocatalysts such as MOFs, g-C_3_N_4_, and TiO_2_, whose ill-defined active-site distributions complicate mechanistic assignment, discrete molecular Co(II) complexes offer a single crystallographically defined coordination environment, enabling direct correlation between structure (SCXRD), electronic state (XPS), and catalytic function. The synthetic accessibility of such complexes has been further expanded by solvothermal in situ coordination chemistry, wherein organic small molecules undergo cyclization and coupling directly within the reaction medium [31,32,33,34]. Metal salts frequently participate in—and are in some cases indispensable to—promoting these transformations, yielding ligand frameworks that would be difficult to obtain through conventional organic synthesis [34]. The resulting complexes typically exhibit broad light absorption profiles, making this strategy well suited to the preparation of photosensitizer complexes for photocatalytic and photovoltaic applications.

Despite these advances, several critical gaps remain unaddressed. First, the overwhelming majority of Co(II) photocatalysts reported to date are either mononuclear molecular complexes or extended coordination polymers. Discrete low-nuclearity clusters in which two Co(II) centers are held in precise geometric proximity by a bridging chromophoric ligand have scarcely been explored for visible-light aerobic oxidation. Second, while MLCT excitation in mononuclear Co(II) systems has been characterized, the cooperative interplay between MLCT and LLCT pathways within a bimetallic unit—and its consequences for O_2_ activation selectivity—has not been systematically interrogated. Third, achieving simultaneously high conversion, sulfoxide chemoselectivity, and catalyst recyclability under purely aerobic, noble-metal-free conditions remains challenging for molecular Co(II) photocatalysts.

Herein, we report a crystalline binuclear cobalt(II) complex (**Co_2_**), in which two CoN_3_O_3_ secondary building units are bridged by photoactive polypyridine linkers to interlock chromophoric and catalytic functions within a single discrete architecture. The assembly absorbs strongly in the blue region and, upon 400 nm LED excitation under ambient air, generates superoxide radicals (O_2_^•–^) that drive the selective aerobic oxidation of thioethers to sulfoxides. Crucially, **Co_2_** outperforms both its constituent Co^II^ salt and the free polypyridine ligand by more than an order of magnitude in yield, underscoring the indispensable role of the preorganized bimetallic environment in sustaining cooperative MLCT/LLCT excitation and productive O_2_ activation. By integrating light-harvesting, charge-transfer, and O_2_-activation functions within a single discrete bimetallic architecture, Co_2_ achieves superoxide-mediated aerobic oxidation of thioethers to sulfoxides with >95% yield and 100% selectivity under 400 nm LED irradiation, while retaining full activity over five consecutive recycling runs. Beyond this specific transformation, the present design illustrates how a low-nuclearity coordination platform can integrate light harvesting, charge separation, and redox catalysis without recourse to precious metals, opening a versatile way to sustainable aerobic transformations driven by visible light.

## 2. Materials and Methods

### 2.1. Synthesis of Co_2_

Synthesis of **Co_2_**. Pyridin-2-ylmethanamine (25 µL, 0.25 mmol), picolinaldehyde (26 µL, 0.25 mmol) and Co(NO_3_)_2_·6H_2_O (146 mg, 0.5 mmol) were dissolved in a 15 mL polytetrafluoroethylene reactor containing 9 mL CH_3_CN. Then, the ampulla was heated at 140 °C for 48 h. After that, the sealed autoclave was subsequently allowed to cool to room temperature naturally over approximately 12 h. The mother liquor was carefully decanted, and the crystalline product was recovered by vacuum filtration. The collected crystals were washed with anhydrous acetonitrile (3 × 3 mL) followed by diethyl ether (2 × 3 mL), and dried at room temperature under ambient atmosphere for 12 h to afford **Co_2_** as large reddish-orange block crystals (170 mg, 85% based on pyridin-2-ylmethanamine). Phase purity of the bulk sample was confirmed by powder X-ray diffraction (PXRD).

### 2.2. Light-Initiated Thioether Oxidation

The photocatalytic oxidation of thioethers was conducted in an airtight Schlenk reactor subjected to irradiation from a 30 W blue light source; the light absorption range of the blue LED is shown in Figure S1. The reaction was carried out with the light source located 1 cm from the outer wall of the transparent Schlenk tube, providing an intensity of 100 mW/cm^2^. A small fan, positioned laterally and directed at the Schlenk tube, was used to keep the reaction at room temperature. In a standard procedure, **Co_2_** (0.05 mmol), 0.2 mmol of sulfide substrate, and 2 mL of methanol (MeOH) were combined within 1 h in the reactor under an ambient atmosphere. The reaction mixture was stirred magnetically at 300 rpm throughout the irradiation period to ensure homogeneous suspension of the solid catalyst and uniform exposure of the reaction mixture to the incident light. Upon completion of the reaction, the resulting mixture was filtered to separate the solid catalyst. The oxidation products were identified and verified by gas chromatography-mass spectrometry (GC-MS). All reported yields were determined by GC-MS analysis using an internal standard calibration method, in which authentic samples of both the sulfoxide product and the corresponding sulfone were used to construct independent calibration curves prior to analysis. This approach allows independent, quantitative verification of both conversion and chemoselectivity. GC-MS analysis of reaction mixtures at the endpoint confirmed the complete consumption of the thioether substrate, with no detectable residual substrate peak above the instrument detection limit. Critically, no chromatographic peak attributable to the sulfone over-oxidation product was observed in any of the substrate scope experiments, confirming that the 100% sulfoxide selectivity is not an artifact of incomplete conversion but reflects a genuine mechanistic preference for the monooxidation pathway. The reported yields are therefore directly calibrated against authentic reference standards and accurately reflect the isolated product distribution.

Reaction condition optimization studies (Figure S1) were conducted using a general screening protocol based on the standard procedure described above, in which a single reaction variable was systematically varied while all other parameters were held constant. For solvent screening (Figure S1a), the following solvents were evaluated as direct replacements for methanol (CH_3_OH, 2 mL), with all other conditions unchanged: acetone, acetonitrile (CH_3_CN), dimethyl sulfoxide (DMSO), tetrahydrofuran (THF), and N,N-dimethylformamide (DMF). For catalyst loading optimization (Figure S1b), the amount of **Co_2_** was varied in the range of 0.015–0.09 mmol with CH_3_OH as solvent and an irradiation time of 1 h. For irradiation time optimization (Figure S1c), the catalyst loading was fixed at 0.03 mmol with CH_3_OH as solvent and irradiation times ranging from 20 to 100 min were evaluated. In all cases, the reaction mixture was stirred magnetically at 300 rpm at 30 °C under ambient air, and the product distribution was analyzed by GC-MS following filtration of the solid catalyst.

The chemical structures of the products were confirmed by comparison with standard chemicals and GC-MS (Agilent Technologies, GC 7890B, MS 5977A, Santa Clara, CA, USA) data. GC equipped with a FID detector (Agilent Technologies, GC 7890B) and an HP-5 5% phenyl methyl siloxane column (30 m × 0.32 mm × 0.5 μm), which was used for the quantifiable measure of (methylsulfinyl)benzene.

### 2.3. EPR Spin-Trapping Measurements

In a standard EPR spin-trapping experiment, **Co_2_** (0.03 mmol) and DMPO (5,5-dimethyl-1-pyrroline N-oxide, 50 mM concentration) were dissolved in CH_3_OH (2 mL) under ambient air atmosphere. After thorough mixing, the solution was immediately transferred into a standard quartz capillary, which was then placed into a quartz EPR sample tube for EPR measurement. Prior to irradiation, a background EPR spectrum was recorded to confirm the absence of pre-existing radical signals. Then, the blue LED light was turned on to irradiate the sample, collecting data simultaneously during irradiation. All spectra were acquired within 5 min of sample preparation to minimize thermal decomposition of the DMPO–O_2_^•–^ adduct. Control experiments performed under identical conditions but in the absence of light, in the absence of **Co_2_**, or under an N_2_ atmosphere in place of ambient air yielded no detectable DMPO spin adduct signals, confirming that photocatalyst, visible-light irradiation, and molecular oxygen are all required for superoxide radical generation. CH_3_OH acts as a quencher of hydroxyl radicals (•OH), so the characteristic peak of hydroxyl radicals was not detected.

## 3. Results and Discussion

### 3.1. Characterization of Crystal Structure

**Figure 1:**
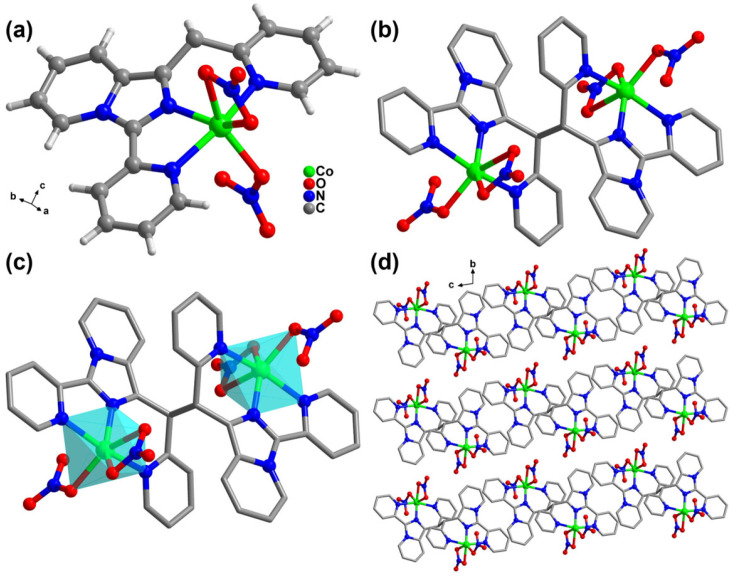
(**a**) The asymmetric unit of **Co_2_**; (**b**–**d**) the binuclear complex of **Co_2_**.

A giant reddish-orange **Co_2_** crystal was synthesized under hydrothermal conditions. The crystal structures are shown in Figure 1 and listed in Table S1. The single-crystal X-ray diffraction (SCXRD) patterns indicate that **Co_2_** crystallizes in the triclinic *P*-1 space group, whose asymmetric unit contains one Co(II) ion, half a **L1** ligand, and two coordinated NO_3_^−^ anions (Figure 1a). The ligand in the unit has an identical coordination environment, wherein the coordinated mode is μ_6_-η^1^:η^1^:η^1^:η^1^:η^1^:η^1^ (Figure 1b). So, the molecular formula is [Co(**L1**)(NO_3_)_4_] (Co_2_) (Figure 1a). The bond length distances of Co-N between Co(II) ions and **L1** ligands are within the limits of 1.995 (2)–2.166 (2) Å, and the bond length distances of Co-O between Co(II) ions and NO_3_^−^ anions are within the limits of 2.086 (2)–2.301 (2) Å (Table S2). The **L1** ligand connects two CoN_3_O_3_ cores (Figure 1c). These binuclear complexes are further organized into a three-dimensional framework through weak interactions (Figure 1d).

**Figure 2:**
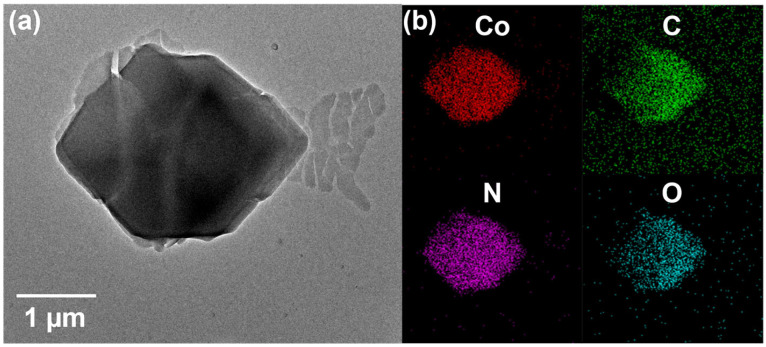
(**a**) The TEM images of **Co_2_**; (**b**) TEM mapping images of **Co_2_**.

**Figure 3:**
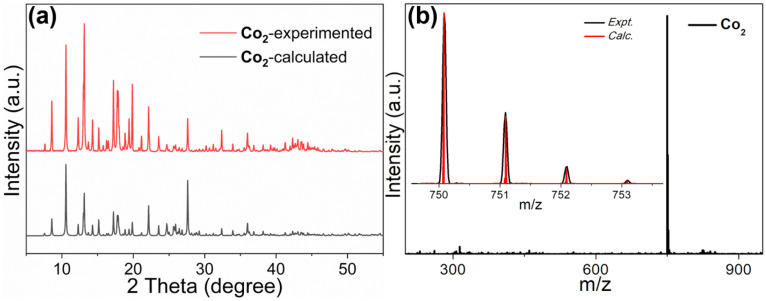
(**a**) The PXRD and (**b**) HRESI-MS of **Co_2_**.

The high-resolution transmission electron microscopy (HRTEM) analysis of **Co_2_** reveals a bulk-like morphology with dimensions of approximately 1.5 µm (Figure 2a). Attempts were made under multiple beam conditions and at the thinnest accessible regions of the crystal edges to resolve lattice fringes or crystal planes by HRTEM. Energy-dispersive X-ray (EDX) analysis of **Co_2_** confirms the presence of C, N, O, and Co elements (Figure 2b), indicating that the samples possess a consistent chemical composition that aligns with the expected crystal structure. The powder X-ray diffraction (PXRD) pattern of **Co_2_** exhibited the same characteristic diffraction peaks, which can be attributed to the corresponding simulated pattern (Figure 3a). The single-crystal solution high-resolution electrospray ionization mass spectrometry of **Co_2_** provided the mass spectrometry data in the positive mode (Figure 3b). At *m*/*z* = 750.08, the highest intensity fragment peak was obtained for the main core, and the molecular ion peak is [Co_2_(**L1**)(NO_3_)_3_]^+^ (calc. 750.08) by fitting. We speculated that the main frame was obtained as the species [Co_2_(**L1**)(NO_3_)_3_]^+^ by losing one NO_3_^−^ anion in methanol solvent.

**Figure 4:**
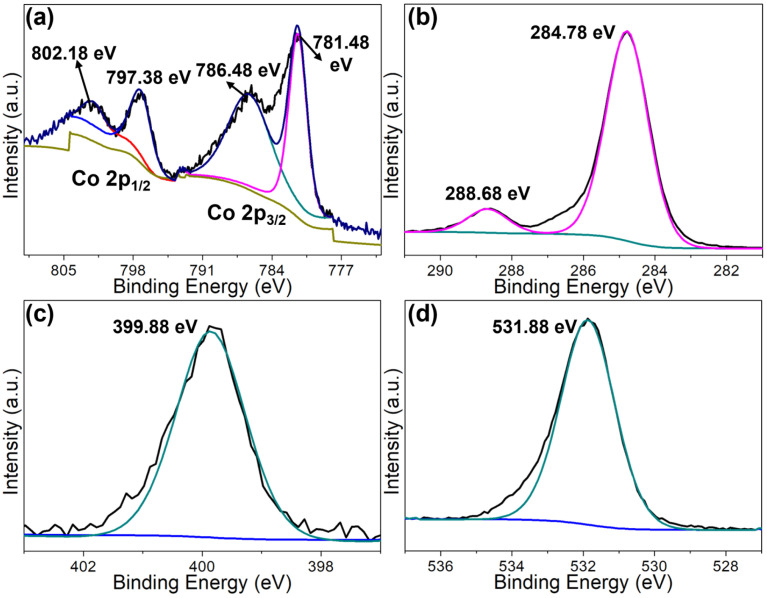
The XPS spectra of Co 2p (**a**), C 1s (**b**), N 1s (**c**) and O 1s (**d**) of **Co_2_**.

The electronic structure and chemical environment of the constituent atoms in **Co_2_** were probed by X-ray photoelectron spectroscopy (XPS). The high-resolution Co 2p spectrum displays a spin–orbit doublet (Figure 4a), with the Co 2p_3/2_ peak resolved into two contributions at 781.48 and 786.48 eV and the Co 2p_1/2_ peak appearing at 797.38 and 802.18 eV. The ca. 16 eV doublet splitting together with the pronounced satellite features above the main peaks is diagnostic of high-spin Co(II) and corroborates the +2 oxidation state assigned by single-crystal X-ray diffraction [35]. In the C 1s region (Figure 4b), two well-resolved signals at 284.78 and 288.68 eV are assigned to aromatic C–C/C=C backbones of the polypyridine framework and to C–N/C=N moieties of the coordinated pyridyl nitrogen, respectively, consistent with the expected ligand connectivity. The N 1s envelope centered at 399.88 eV (Figure 4c) is characteristic of pyridyl nitrogen engaged in dative bonding to Co(II), while a single O 1s component at 531.88 eV (Figure 4d) reflects the carboxylate oxygens of the CoN_3_O_3_ secondary building unit. These binding-energy signatures verify that the as-synthesized **Co_2_** retains the coordination environment determined crystallographically and is free of detectable surface oxidation or impurities.

**Figure 5:**
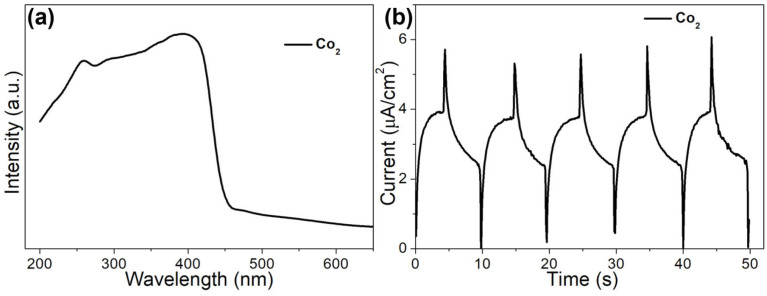
(**a**) The UV-Vis DRS and (**b**) transient photocurrent of **Co_2_**.

The optical response of **Co_2_** was evaluated by ultraviolet–vis diffuse reflectance spectroscopy (Figure 5a). The complex exhibits panchromatic absorption spanning 200–700 nm, with the dominant intensity concentrated below 450 nm and a long, gradually decaying tail extending into the visible region. The high-energy bands are attributable to π–π* and n–π* transitions localized on the polypyridine chromophore, whereas the lower-energy shoulder originates from ligand-to-metal charge-transfer (LMCT) and d–d transitions at the Co(II) centers [36]. This spectral profile establishes **Co_2_** as a competent absorber of 400 nm light and rationalizes its selection as a candidate for blue-light-driven photocatalysis. To assess whether the captured photons translate into separated charge carriers, transient photocurrent measurements were performed under chopped-light illumination (Figure 5b). **Co_2_** generates a prompt, reproducible photocurrent response that switches on and off in synchrony with the light pulses across multiple cycles, demonstrating efficient exciton dissociation and directional charge migration at the electrode interface. Such behavior is a prerequisite for productive single-electron transfer to molecular O_2_, and provides a direct mechanistic link between the absorption properties documented above and the superoxide-driven catalysis discussed in the following sections.

### 3.2. Photocatalytic Properties

To evaluate the photocatalytic performance of **Co_2_**, the oxidation of thioether was selected as a model reaction under blue LED light irradiation. The reaction did not proceed when catalyst-free or Co(NO_3_)_2_·6H_2_O was added to the thioether oxidation system (Table 1, entries 1 and 2). When the **L1** ligand was added to the thioether oxidation system, a 37% yield of (methylsulfinyl) benzene could be obtained (Table 1, entry 3), presumably driven by ligand-centered excitation. However, when **Co_2_** was employed as the photocatalyst, it significantly promoted the reaction, achieving a yield of 99% within 1 h (Table 1, entry 4). The dramatic enhancement in catalytic activity upon coordination can be rationalized by the emergence of additional charge-transfer pathways in **Co_2_** that are absent in the free ligand or the bare metal salt. Specifically, the assembly of **L1** with cobalt enables ligand-to-ligand charge-transfer (LLCT) transitions, in which photoexcitation drives electron density redistribution among the coordinated ligands, thereby generating reactive excited states with prolonged lifetimes. Concurrently, metal-to-ligand charge-transfer (MLCT) transitions, wherein the cobalt center donates electron density to the π* orbitals of **L1** upon light absorption, provide an additional low-energy excitation channel that is well-matched with blue LED irradiation. The synergistic interplay between LLCT and MLCT pathways in **Co_2_** thus substantially broadens the accessible excited-state manifold and facilitates more efficient photoinduced electron transfer to molecular oxygen, ultimately driving the thioether oxidation to near-quantitative conversion.

With the photocatalytic activity of **Co_2_** established, we next sought to define the optimal reaction conditions by systematically varying the solvent, catalyst loading, and irradiation time (Figure S3). Solvent screening revealed a pronounced medium dependence. Polar aprotic solvents commonly employed in oxidation reactions—including acetone, acetonitrile, DMSO, tetrahydrofuran, and DMF—all delivered only trace amounts of (methylsulfinyl) benzene, whereas methanol consistently afforded the highest conversion (Figure S3a). The superior performance of methanol is plausibly traced to its capacity to solubilize both substrate and molecular oxygen while stabilizing the polar transition state leading to the sulfoxide. The optimal amount of photocatalyst was determined to be 0.05 mmol (Figure S3b). The yield of (methylsulfinyl)benzene increased linearly with prolonged irradiation time (Figure S3c), reaching a maximum yield of 99% after 1 h of reaction without onset of over-oxidation to the sulfone. To assess whether the photocatalytic performance of **Co_2_** translates beyond the analytical-scale reaction, a gram-scale experiment was conducted by proportionally increasing 30 times of catalyst and substrate while maintaining all other reaction parameters (methanol solvent, ambient air, 30 W blue LED, 30 °C, 1 h). The sulfoxide product was obtained in 94% isolated yield of 1.18 g after simple filtration of the catalyst and evaporation of the solvent, with no detectable sulfone byproduct by GC-MS analysis. The catalyst was quantitatively recovered by filtration and reused directly in the subsequent run without any reactivation treatment. This result confirms that the high yield and chemoselectivity observed at the analytical scale are preserved upon reaction scale-up, and that the simple workup procedure—filtration and evaporation, without column chromatography—is compatible with practical synthetic application.

To examine the generality of the **Co_2_**-catalyzed transformation across a structurally diverse panel of aryl methyl sulfides bearing substituents of contrasting electronic character (Table 2). The catalyst tolerated the full series, delivering the corresponding sulfoxides as the dominant products with no detectable over-oxidation to sulfones, which testifies to the robustness of the active site under aerobic photocatalytic conditions. Sulfides decorated with a –Cl electron-withdrawing group were converted markedly faster and in higher yield than the –Me electron-donating counterpart. This polarity dependence is opposite to that expected for an electrophilic oxygen-transfer mechanism but is fully consistent with a superoxide-mediated single-electron pathway, in which the sulfide is first oxidized to a sulfur-centered radical cation [ArSR]^•+^. Electron-poor aryl rings raise the oxidation potential of the sulfur lone pair only modestly while stabilizing the ensuing radical cation through conjugation with the electron-withdrawing substituent, thereby lowering the kinetic barrier to its formation. To further confirm the identity of the oxidation product and unambiguously exclude the formation of over-oxidized byproducts, the isolated product obtained from the photocatalytic oxidation of thioanisole under standard conditions was characterized by both ^1^H NMR and ^13^C NMR spectroscopy (Supporting Information at the end). The ^1^H NMR spectrum displays two sets of signals: a multiplet in the aromatic region integrating for five protons (δ 7.45–7.53 ppm, 5H, ArH), and a singlet at δ 2.71 ppm integrating for three protons (3H, S–CH_3_), with an integrated ratio of 5:3 fully consistent with the expected structure of methyl phenyl sulfoxide (PhS(O)CH_3_). Critically, no signal attributable to the methyl group of methyl phenyl sulfone (PhS(O)_3_CH_3_), which would appear at δ 3.05 ppm in ^1^H NMR, was detected, directly confirming the absence of over-oxidation to the sulfone under the photocatalytic conditions employed. The ^13^C NMR spectrum exhibits four resolved signals at δ 146.88, 131.20, 129.17, 124.07 ppm assigned to the aromatic carbons of the phenyl ring, and a signal at δ 43.98 ppm corresponding to the S–CH_3_ carbon, in excellent agreement with the reported chemical shifts for authentic methyl phenyl sulfoxide. No additional carbon signals attributable to sulfone or other byproducts were observed. The ^1^H and ^13^C NMR data unambiguously confirm that methyl phenyl sulfoxide is the sole organic product of the photocatalytic reaction, fully corroborating the 100% chemoselectivity determined by GC–MS analysis.

### 3.3. Photocatalytic Mechanism Studies

**Figure 6:**
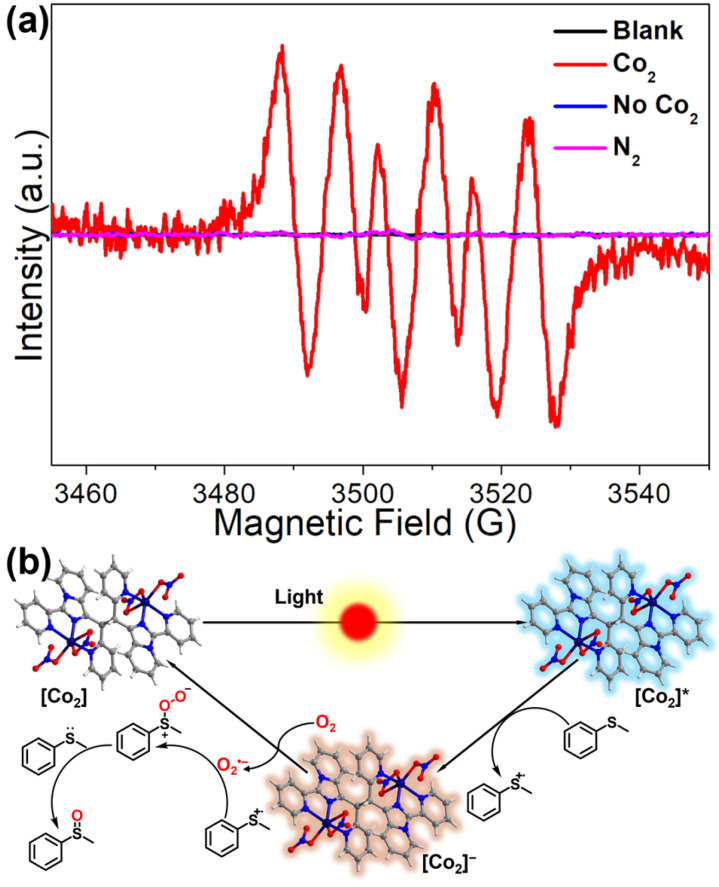
(**a**) EPR spectrum peak of the O_2_^•−^ radical trapped by DMPO; (**b**) The proposed mechanism.

The chelating coordination of the polypyridine ligand **L1** around each Co(II) center serves a dual structural and electronic role. It imposes a rigid, six-coordinate CoN_3_O_3_ geometry that suppresses ligand dissociation under turnover, and it establishes a low-lying MLCT manifold that channels photoexcitation away from the metal centers and onto the π-accepting polypyridine framework. By delocalizing the photogenerated hole onto the ligand, this MLCT pathway shields the Co(II) centers from net reduction during catalysis and preserves the +2 oxidation state across multiple turnovers. Direct photophysical evidence for this MLCT-mediated charge-transfer process was obtained from steady-state photoluminescence (PL) measurements recorded for Co_2_ and free **L1** under identical excitation conditions (λex = 380 nm) in CH_3_OH at room temperature (Figure S4). Free **L1** exhibits a well-defined emission band centered at approximately 437 nm, assigned to radiative decay from the ligand-centered π–π* excited state. Upon coordination in **Co_2_**, the emission intensity is substantially quenched relative to free **L1** under equivalent concentration conditions, confirming that the residual emission in **Co_2_** originates exclusively from the coordinated polypyridine chromophore. This pronounced quenching is consistent with efficient intramolecular deactivation of the ligand-centered excited state via MLCT, in which photoexcited electron density on the polypyridine π* manifold is transferred to the Co(II) d orbitals. To verify the identity of the active oxidant, electron paramagnetic resonance (EPR) measurements were carried out in the presence of 5,5-dimethyl-1-pyrroline N-oxide (DMPO) as a spin trap [37]. Irradiation of an aerated reaction mixture containing **Co_2_** produced the diagnostic four-line DMPO–O_2_^•–^ adduct signal with a characteristic intensity ratio of 1:1:1:1 and the expected hyperfine coupling constants (Figure 6a), unambiguously establishing the O_2_^•–^ radical as the dominant reactive oxygen species. No appreciable signal was detected in the absence of light, of catalyst, or of O_2_, confirming that all three components are required for radical generation (Figure 6a).

On the basis of these observations, together with the spectroscopic and electrochemical evidence presented in the preceding sections and in agreement with reported mechanisms for related Co(II)–complex photocatalysts [38,39], we propose the catalytic cycle depicted in Figure 6b. Blue-light absorption promotes **Co_2_** to its MLCT-derived excited state, [**Co_2_**], in which the photogenerated electron resides predominantly on the polypyridine π manifold while the hole is localized on the cobalt-coordinated sulfur of the bound thioether through inner-sphere interactions. Single-electron transfer from the substrate to this hole affords the sulfur-centered radical cation [ArSMe]^•+^ together with the one-electron-reduced photocatalyst [**Co_2_**]^•–^. The latter is rapidly re-oxidized by dioxygen, regenerating the resting-state **Co_2_** and releasing O_2_^•–^—a step that simultaneously closes the photoredox loop and accounts for the EPR signature described above. Nucleophilic addition of O_2_^•–^ to [ArSMe]^•+^ then yields a transient persulfoxide-type intermediate (ArS(OO^−^)Me), which oxidizes a second equivalent of thioether through oxygen-atom transfer to deliver two molecules of the (methylsulfinyl)benzene product per photocatalytic turn. This bifurcated pathway—electron transfer at the Co(II) center coupled with oxygen activation at the molecular periphery—rationalizes both the substantial rate enhancement over the mononuclear and ligand-only controls and the high selectivity for sulfoxide over sulfone observed throughout the substrate scope.

As shown in Table S3, among thioether oxidation reactions with cluster photocatalysts, **Co_2_** demonstrates several distinct advantages (Entry 1). Relative to noble-metal references (Entries 2–3), it achieves equivalent yield (99%) and chemoselectivity (100%) using exclusively earth-abundant Co, eliminating the cost and scarcity constraints of ruthenium-based catalysts [40,41]. Compared to earth-abundant metal-oxo cluster systems (Entries 4–6) [38,42,43], **Co_2_** requires substantially shorter reaction times (1 h vs. 6–12 h) and employs ambient air rather than stoichiometric H_2_O_2_ as the terminal oxidant, conferring a superior atom-economy and sustainability profile. Relative to heterogeneous MOF and POMOF systems (Entries 7–8), **Co_2_** operates in methanol with simpler workup and comparable recyclability [44,45]. Crucially, as the only earth-abundant 3d transition-metal coordination cluster in this comparison, the discrete binuclear architecture of **Co_2_** provides a crystallographically defined CoN_3_O_3_ active site that enables direct structure–activity correlation and unambiguous mechanistic interrogation, a level of structural precision that is inaccessible in the higher-nuclearity Ce and Ti cluster systems.

## 4. Conclusions

In conclusion, we have successfully obtained a discrete binuclear cobalt(II) complex (**Co_2_**) assembled solvothermally from CoN_3_O_3_ secondary building units bridged by photoactive polypyridine linkers, which functions as a noble-metal-free photocatalyst for the visible-light-driven aerobic oxidation of thioethers. The two Co(II) centers each retain a pair of labile NO_3_^−^ anions that desorb under turnover to expose accessible coordination sites, providing direct contact between the redox-active metals and the incoming substrate. Coupled with the strong blue-light absorption and efficient charge separation conferred by the polypyridine framework, this architecture integrates light-harvesting, substrate activation, and oxygen reduction within a single binuclear unit. Mechanistic interrogation by EPR spin-trapping, photocurrent measurements, and substrate-electronic-effect analysis converges on a superoxide-mediated single-electron-transfer cycle, in which MLCT excitation gates electron flow from the substrate to dioxygen while preserving the Co(II) oxidation state across consecutive turnovers. Under 400 nm LED irradiation in air, **Co_2_** selectively converts a structurally diverse panel of aryl methyl sulfides to the corresponding sulfoxides in yields exceeding 95%, with no detectable over-oxidation to the sulfone, and retains undiminished activity over at least five recycling runs. This work demonstrates how cooperative photophysical and redox activity can be embedded within a low-nuclearity coordination framework to eliminate the need for precious metals.

## Figures and Tables

**Table 1:** Photocatalytic oxidation of thioether over different photocatalysts.

Entry	Photocat.	*Yield* (%)	*Sel.* (%)
1	None	0	0
2	Co(NO_3_)_2_·6H_2_O	0	0
3	**L1**	37	100
4	**Co_2_**	99	100

Reaction conditions: 0.05 mmol photocatalyst, 0.3 mmol thioanisole, 2 mL methanol, air, 30 W blue LED lamp, 1 h, 30 °C. The yield was determined by GC-MS.

**Table 2:** Substrate scopes of photocatalytic aerobic oxidation over **Co_2_**.

Entry	Product	*Con.* (%)	*Sel.* (%)
1 ^a^	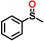	99	99
2 ^a^	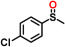	99	99
3 ^a^	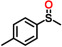	99	99
4 ^b^	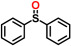	95	99
5 ^b^	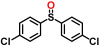	99	99
6 ^b^	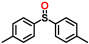	87	99

Reaction conditions: ^a^ 0.05 mmol **Co_2_**, 0.3 mmol sulfides, 2 mL methanol, air, 1 h, 30 W blue LED lamp, 30 °C; ^b^ 0.05 mmol **Co_2_**, 0.15 mmol sulfides, 2 mL methanol, air, 1 h, 30 W blue LED lamp, 30 °C. The yield was determined by GC-MS.

## Data Availability

The original contributions presented in this study are included in the article/Supplementary Materials. Further inquiries can be directed to the corresponding authors.

## Supplementary Materials

The following supporting information can be downloaded at: https://www.mdpi.com/article/10.3390/nano16130835/s1, Materials and Characterization; Single-crystal X-ray crystallography; Crystallographic data; Bond lengths and angles; Elemental map; Photocatalyst characterization. References [38,40,41,42,43,44,45,46] are cited in the supplementary materials.
