# Supplementary material for: Blue-Light-Driven Aerobic Oxidation via ROS-Generating Binuclear Cobalt(II) Complex Photocatalyst
Source: Nanomaterials (Basel). 2026 Jul 7;16(13):835. doi: 10.3390/nano16130835 (PMC13363593; doi:10.3390/nano16130835)

Blue-light-driven aerobic oxidation via ROS-generating binu-clear Cobalt(II) complex photocatalyst

Yuhao Mu [1], Zhuang Miao [1], Rong Zhang [1*], Xiong-Feng Ma [1] and Zhipeng Xie [2*]

[1] College of Engineering, Xi'an International University, Xi'an 710077, China

[2] Department of Chemistry, The Chinese University of Hong Kong, New Territories 999077, Hong Kong, P. R. China

* Correspondence: zpxie@hku.hk (Z. Xie), xaiu24206@xaiu.edu.cn (R. Zhang)

1. Materials and Characterization

All reagents and solvents are commercially available (Beijing innoChem Science & Technology Co. Ltd. China), and they were further dried by the vacuum rotary evaporator to remove all traces of water and then stored in the air-filled glovebox. The 20 mL Schlenk tube charged with a magnetic stirrer, which was used as the reactor in the thioether oxidation reaction. The light was provided by a 30 W blue LED light.

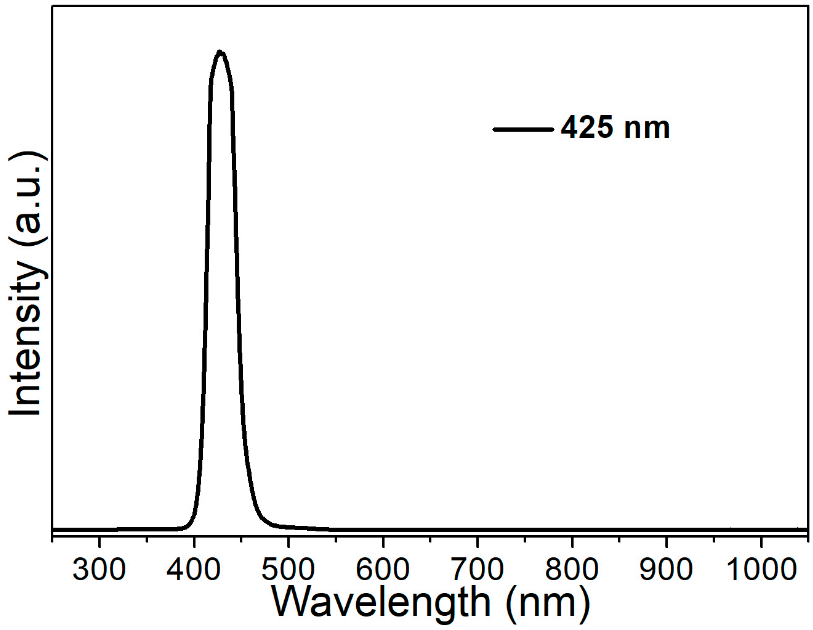

Figure S1. The light absorption range of blue LED.

The transmission electron microscopy (TEM) images were measured on a Thermo Scientific Talos F200S G2 microscope (Massachusetts, USA) with an acceleration voltage of 200 kV. A Varian Cary 500 UV-Vis spectrophotometer (California, USA) was used to record the UV-Vis diffuse reflectance spectra (DRS) of various solid samples. The photoelectrochemical characterization was performed on a Metrohm-Autolab AUT302N Electrochemical workstation (Jiangsu, China). Photocurrent measurements were carried out in a typical three-electrode configuration with an Ag/AgCl electrode, a coiled Pt wire as the reference and counter electrode, respectively. The electron paramagnetic resonance (EPR) experiments were carried out on Bruker A300 instrument (Germany) operating in the X-band at room temperature. ^{1}H and ^{13}C spectra were recorded on a Bruker UltraShield Plus Avance III (500 MHz, 126 MHz, respectively, Germany). Peaks were referenced to residual solvent ($CDCl_3$).

2. Single-crystal X-ray crystallography

Diffraction data for all complexes were measured on a Bruker SMART CCD diffractometer (Mo Kα radiation and λ = 0.71073 Å) in Φ and ω scan modes. All structures were solved by direct methods, followed by difference Fourier syntheses, and then refined by full-matrix least-squares techniques on F^2 using SHELXL.[1] All other non-hydrogen atoms were refined with anisotropic thermal parameters. Hydrogen atoms were placed in the calculated position and refined in the isotropic direction using a riding model. Table S1 summarizes X-ray crystallographic data and refinement details for the complexes. The CCDC reference numbers are 2500233 for **Co_2**.

Table S1. Crystallographic data of the complex **Co_2**.

**Complex**	**Co_2**
Formula	$C_{36}H_{26}Co_2N_{12}O_{12}$
Formula weight	936.55
T (K)	100.00 (10)
Crystal system	Triclinic
Space group	P-1
a (Å)	7.2755 (4)
b (Å)	10.9867 (6)
c (Å)	11.9896 (7)
α (°)	94.857 (5)
β (°)	99.942 (5)
γ (°)	108.699 (5)
V (Å^3)	884.03 (9)
Z	1
D_c(g cm^{-3})	1.759
μ (mm^{-1})	8.119
Reflns coll.	6570
Unique reflns	3430
R_{int}	0.0345
$^aR_1[I \geq 2\sigma(I)]$	0.0435
$^bwR_2$(all data)	0.1114
GOF	1.045

$^aR_1 = \Sigma||F_o|-|F_c||/\Sigma|F_o|$, $^bwR_2 = [\Sigma w(F_o^2-F_c^2)^2/\Sigma w(F_o^2)^2]^{1/2}$

Table S2. Selected bond lengths (Å) and angles (°) of complex **Co2**.

**Bond lengths (Å)**					
Co1—N1	2.166 (2)	Co1—N3	1.995 (2)	Co1—N4	2.155 (2)
Co1—O1	2.301 (2)	Co1—O2	2.127 (2)	Co1—O4	2.086 (2)
**Bond angles (°)**					
O4—Co1—O2	83.82 (8)	O2—Co1—N4	104.49 (9)	N3—Co1—N4	88.09 (9)
O4—Co1—O1	141.09 (8)	O2—Co1—N1	94.13 (9)	N3—Co1—N1	76.37 (9)
O4—Co1—N4	94.76 (8)	N3—Co1—O4	133.10 (8)	N4—Co1—O1	91.61 (8)
O4—Co1—N1	88.58 (8)	N3—Co1—O2	140.49 (9)	N4—Co1—N1	161.32 (9)
O2—Co1—O1	57.47 (8)	N3—Co1—O1	85.38 (8)	N1—Co1—O1	97.30 (8)
Symmetry code: (i) -*x*+1, -*y*+1, -*z*+1.					

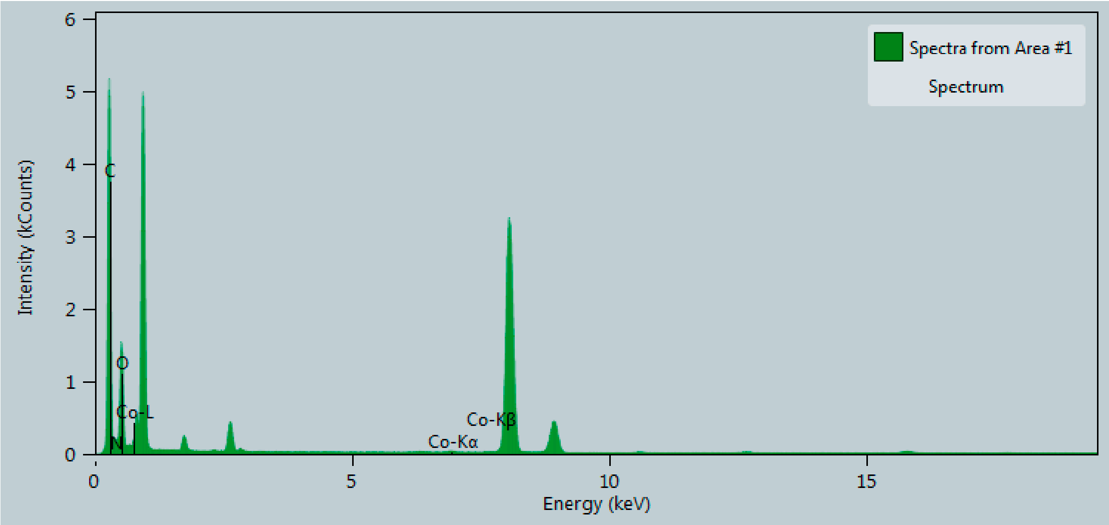

Figure S2. The elemental map of Co2.

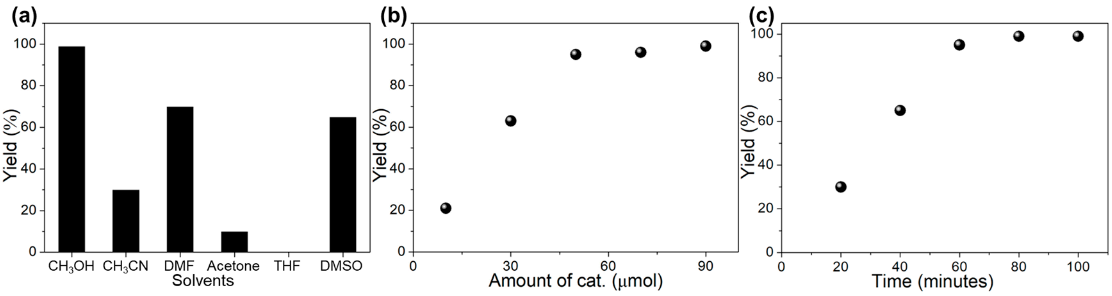

Figure S3. (a) Yields of (methylsulfinyl)benzene in different solvents for **Co_2**; (b) the influence of photocatalyst amounts and (c) irradiation times of **Co_2** on the yield of (methylsulfinyl)benzene.

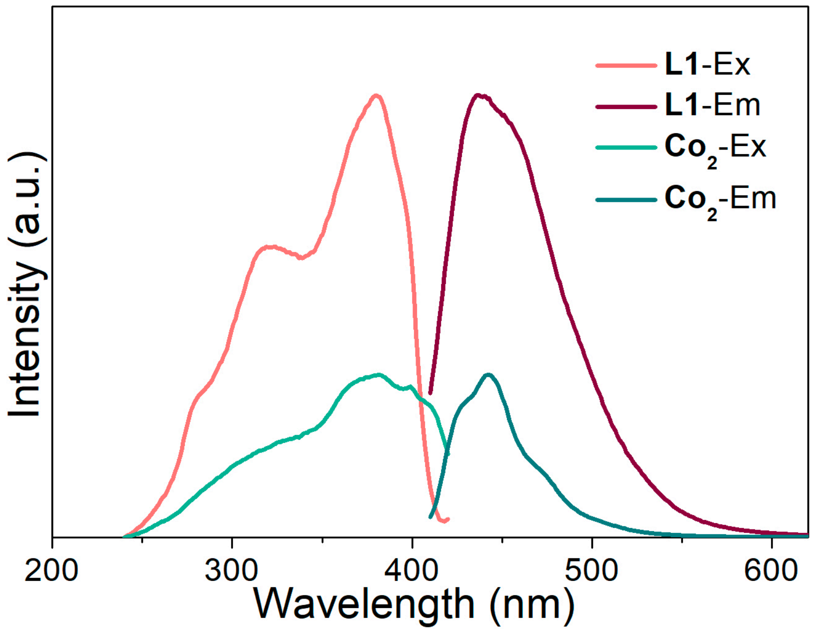

Figure S4. The PL spectra of **L1** and **Co_2**.

Table S3. Comparison of **Co_2** with representative catalysts for photocatalytic aerobic oxidation of thioethers to sulfoxides

Entry	Catalyst	Oxidant	Time (h)	Yield (%)	Sel. (%)	Ref.
1	**Co_2**	Air	1	99	100	This work
2	Ru–Cu cluster	Air	N.R.	>95	>95	2
3	cis-$[Ru(bipy)_2]^{2+}$	O_2	N.R.	>90	>95	3
4	Ce16 cluster	O_2	12	>90	>95	4
5	Ti-oxo/phen cluster	O_2	8	>85	>90	5
6	Ti-oxo cluster	H_2O_2	6	>90	>95	6
7	Zr-MOF	O_2	3	>95	>95	7
8	$CoBW_{12}$–TPT	O_2	4	>90	>95	8

1. Sheldrick, G. M. *Acta Crystallogr., Sect. C: Struct. Chem.* **2015**, 71, 3-8.

2. Chao, D.; Zhao, M. Robust Cooperative Photo-oxidation of Sulfides without Sacrificial Reagent under Air Using a Dinuclear RuII – CuII Assembly. *ChemSusChem* **2017**, 10, 3358-3362.

3. Cuéllar, E.; Diez-Varga, A.; Torroba, T.; Domingo-Legarda, P.; Alemán, J.; Cabrera, S.; Martín-Alvarez, J. M.; Miguel, D.; Villafañe, F. Luminescent cis-Bis(bipyridyl)ruthenium(II) Complexes with 1,2-Azolylamidino Ligands: Photophysical, Electrochemical Studies, and Photocatalytic Oxidation of Thioethers. *Inorg. Chem.* **2021**, 60, 7008–7022.

4. Lv, X.; Zhao, X.-l.; Zhao, Q.; Zheng, Q.; Xuan, W. Cerium-oxo clusters for photocatalytic aerobic oxygenation of sulfides to sulfoxides. *Dalton Trans.* **2022**, 51, 8949-8954.

5. Liao, L.-R.; Zheng, D.-C.; Ou, P.-X.; Zhao, Q.-X.; Xuan, W.-M.; Zheng, Q. π-conjugated chromophore functionalized high-nuclearitytitanium-oxo clusters containing structural unit of anatasefor photocatalytic selective oxidation of sulfides. *Rare Met.* **2024**, 43, 1736–1746.

6. Dai, L.-F.; Liu, X.-R.; Tian, Y.-Q.; Yi, X.-Y.; Liu, C. Auxiliary Carboxylate-Induced Assembly of Calix[6]arene-Polyoxotitanate Hybrid Systems with Photocatalytic Activity in the Oxidation of Sulfides. *Inorg. Chem.* **2023**, 62, 6047–6054.

7. Chen, J.-Q.; Zhang, K.-Y.; Zhang, X.-D.; Huang, Z.-Q.; Deng, H.; Zhao, Y.; Shi, Z.-Z.; Sun, W.-Y. A Green Environmental Protection Photocatalytic Molecular Reactor for Aerobic Oxidation of Sulfide to Sulfoxide. *Chem.-Eur. J.* **2024**, 30, e202303725

8. Liu, X.; Si, C.; Xu, J.; Sun, H.; Li, J.; Han, Q. Constructing a Polyoxometalate-Based Metal–Organic Framework for Photocatalytic Oxidation of Thioethers to Sulfoxides Utilizing In Situ-Generated Superoxide Radicals. *Inorg. Chem.* **2025**, 64, 1263–1271.

NMR data for **1**.

After reaction, solvent not participating in the reaction was removed under reduced pressure. The crude products were purified by SiO_2/Al_2O_3 column chromatography using acetic ether and petroleum ether (V:V=1:1) as an eluent so that the white solids acylamide derivatives can be obtained. 0.25 mm silica gelcoated glass plates were used in the thin layer chromatography (TLC).

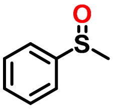

: ^{13}C NMR (151 MHz,) δ 146.88, 131.20, 129.75, 124.07, 43.87, 43.80, 43.78, 43.73. ^{1}H NMR (600 MHz,) δ 7.65, 7.64, 7.63, 7.54, 7.53, 7.52, 7.50, 7.49, 7.47, 2.70, 2.69, 2.68.

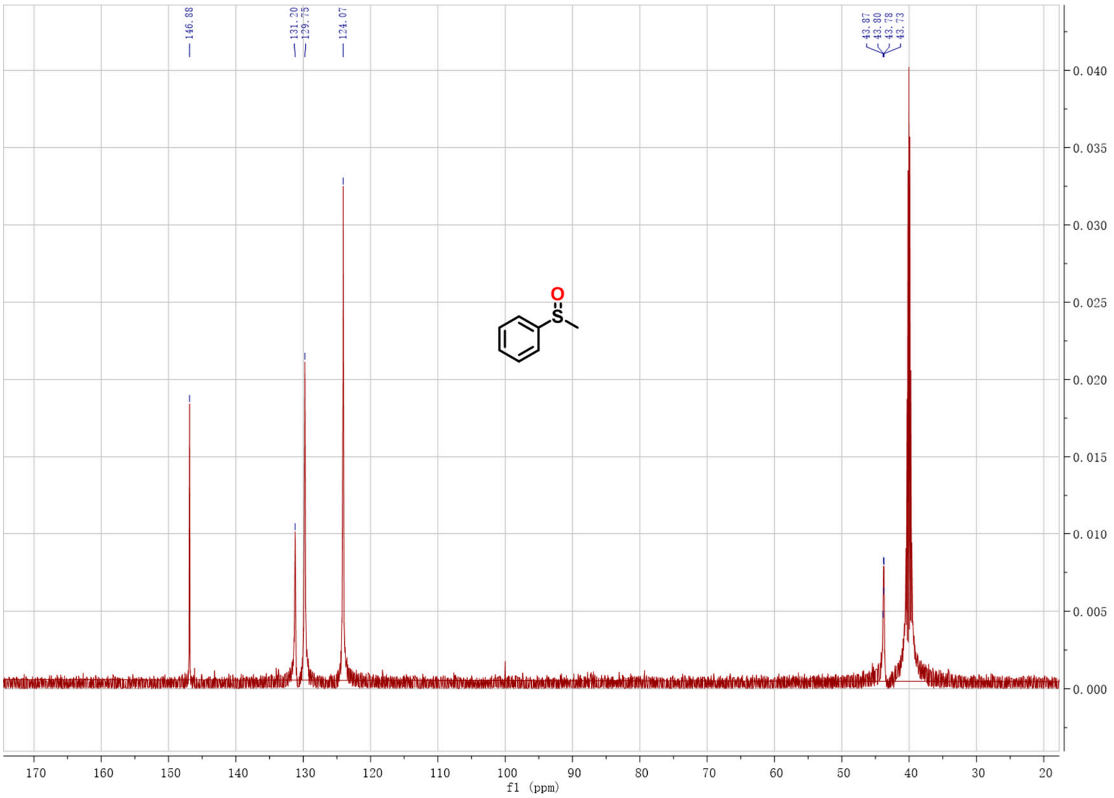

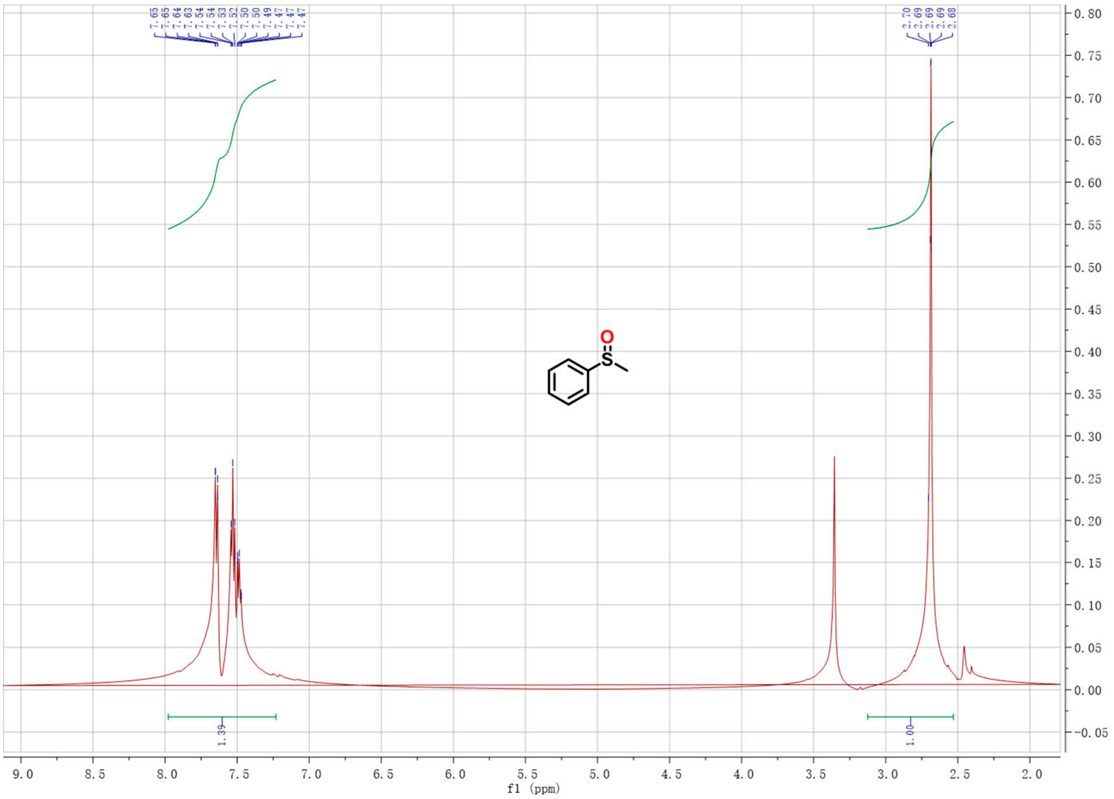

Supplement: Supplementary file 1 [file nanomaterials-16-00835-s001.zip › nanomaterials-4373150-supplementary.pdf]
